# Physiological and Nutritional Responses of Pear Seedlings to Nitrate Concentrations

**DOI:** 10.3389/fpls.2018.01679

**Published:** 2018-11-20

**Authors:** Guodong Chen, Li Wang, Musana R. Fabrice, Yanan Tian, Kaijie Qi, Qian Chen, Peng Cao, Peng Wang, Shaoling Zhang, Juyou Wu, Shutian Tao

**Affiliations:** Center of Pear Engineering Technology Research, State Key Laboratory of Crop Genetics and Germplasm Enhancement, College of Horticulture, Nanjing Agricultural University, Nanjing, China

**Keywords:** rootstock, root system architecture, physiological performance, nutrients, elements

## Abstract

Nitrogen (N) is an important element for plant growth, and a suitable N supply is crucial to ensure optimal yields from fruit trees. Frequently, application of N fertilizers to fruit trees is often excessive, which not only leads to environmental pollution, but also reduces the output from fruit trees through N toxicity. To evaluate the effects of different concentrations of nitrate on plant growth, root-morphological traits, and other nutritional element’s responses in pear, pear seedlings (*Pyrus betulifolia* Bunge) were treated with five levels of N. Both N-deficiency and an excess of N inhibited the growth and development of pear rootstocks. However, different visible symptoms were observed among treated leaves and roots. Leaf yellowing, the stimulation of root elongation, a decrease in nitrate reductase activity and chlorophyll content were observed under N-deficiency conditions. On the other hand, dark green leaves accompanied by coking, the suppression of root elongation, and a decrease in nitrate reductase activity and chlorophyll content were displayed under regimes of excess N. In addition, not only the N content, but also the content of other mineral nutrients was influenced by nitrate treatments. Taken together, these results suggested that a careful choice of N fertilizer supply is crucial to ensure normal growth and development in pear trees.

## Introduction

Nitrogen is one of the most abundant elements on Earth (Crawford and Glass, 1998; Hirsch and Sussman, 1999). However, the level of biologically available N often fails to meet crop requirements in agricultural systems having aerobic soils, in which nitrate is easily leached under high rainfall conditions (Fernández-Escobar et al., 2004; Giles, 2005; Masclaux-Daubresse et al., 2010; Canales et al., 2014). Thus, N is still one of the major factors limiting crop yields.

It is well known that N deficiencies limit plant growth and development, thereby reducing crop yields. The major causal effects of N-deficiency are reductions in protein synthesis and the chlorophyll content, resulting in a reduced accumulation of photosynthetic products (Daughtry et al., 2000; Hirel et al., 2011; Alvarez et al., 2012). On the other hand, the excessive use of N fertilizers not only requires a greater investment from farmer for a reduced crop yield, but also leads to N contamination of the groundwater (Jaynes et al., 2001). Therefore, the establishment of a reasonable N supply to crops is fundamental to balance gains in crop yields against the increasing costs of N fertilizer and the need to minimize environmental perturbations, especially water quality.

Previous studies have shown that RSA plays a critical role in plant adaptation to external, environmental fluctuations. Plant roots can exhibit different physiological characteristics when grown under different N concentrations (Lynch, 1995; Osmont et al., 2007). In addition, ${\text{NO}}_{3}^{-}$ deficiency causes an increase in root hair length and density in spinach (Lynch, 1995; Gruber et al., 2013), and to a lesser extent in tomato (Foehse and Jungk, 1983). Furthermore, total root size, primary root length, and first- and second-order LR numbers significantly increased under conditions of nitrate starvation in wheat (Sinha et al., 2015). In contrast, excess N causes a decrease in primary root length and LR elongation in *Arabidopsis* (Zhang et al., 1999; Linkohr et al., 2002). Taken together, we may conclude that the availability of different concentrations of N can evoke distinct effects on the RSA.

In China, pear is the third most important fruit crop, and China leads the world in pear production and has the largest area of cultivation dedicated to this crop (Wu et al., 2013). The production of pear is significantly influenced by different levels of N supply. However, little is known about its adaptability to a deficient or excessive N level. Thus, the objectives of this study were to evaluate the effects of excessive and deficient N levels on growth, root-morphological traits, root activity, nitrate reductase activity, and the contents of N and other mineral nutritional elements, as well as to investigate the relationship among physiological performance, physiological activity, and mineral nutrients. These results highlight the necessity to determine the optimal supply of N fertilizer to optimize yields from pear and other fruit tree species.

## Materials and Methods

### Plant Materials and Treatment

Birch-leaf pear (*Pyrus betulifolia* Bunge) were used in these experiments. First, seeds were soaked with water for 24 h and surface sterilized for 15 min in a 3% sodium hypochlorite solution and then rinsed repeatedly by deionized water. Seeds were mixed with sand (5–10% moisture content) to a volume ratio of 1:4, placed in a mesh bag and placed at the bottom of a foam box with moist sand and then transferred to 4°C for stratification for 40 days. They were then transferred into a growth chamber for 2 days until seed germination, before being sown into 5 × 10 hole plastic containers filled with vermiculite. After 14 days, 45 seedlings of uniform size were selected, and then seedlings were solution-cultured in a plastic barrel covered with black KT board (Surin Flower Company, China) under growth chamber conditions. Seedlings were precultured in 1/2 Hoagland’s nutrient solution for 1 week until the growth of new roots, and then placed in 1× strength Hoagland’s nutrient solution containing ${\text{NO}}_{3}^{-}$ at either 16 (control), 0, 4, 32, or 64 mM. Nine seedlings were used per treatment, with three replications. The seedlings were randomly arranged and cultured for 5 weeks, when typical symptoms of deficient or excess N became apparent.

The experiment was carried out in a growth chamber (Jiangnan Instrument, Ningbo, China) with a light/dark regime of 14/10 h, at 28/22°C, and 75% relative humidity, with a light intensity of 800 μmol m^-2^ s^-1^ of photosynthetically active radiation. Full strength Hoagland’s nutrient solution was used as the base for deficient or excess N nutritive solutions, which contained 14 mM NaNO_3_, 1 mM Ca(NO_3_)_2_⋅4H_2_O, 1 mM KH_2_PO_4_, 2 mM MgSO_4_⋅7H_2_O, 0.83 mg⋅L^-1^ KI, 6.2 mg⋅L^-1^ H_3_BO_3_, 22.3 mg⋅L^-1^ MnSO_4_, 8.6 mg⋅L^-1^ ZnSO_4_, 0.025 mg⋅L^-1^ CuSO_4_, 0.025 mg⋅L^-1^ CoCl_2_, 0.25 mg⋅L^-1^ Na_2_MoO_4_, and 50 μM Fe-EDTA (Hoagland and Arnon, 1950) (Table 1). The solution was ventilated for 30 min every 3 h by the combined action of a timer (Pinyi AL-06, China) and ventilation pump (SUNSUN ACO, China), and replaced twice a week. The pH of all nutrient solutions was adjusted to 5.8 with 0.1 M KOH.

**Table 1:** Composition of the nutrient solution used for the experiment.

Composition		0 mM	4 mM	16 mM	32 mM	64 mM
Macroelement (mM)	KH_2_PO_4_	1	1	1	1	1
	MgSO_4_⋅7H_2_O	2	2	2	2	2
	KCl	5	5	5	5	5
	Ca(NO_3_)_2_⋅4H_2_O	0	0.5	1	1	1
	CaCl_2_	4	2	0	0	0
	NaNO_3_	0	0	14	30	62
Microelement (mg/L)	KI	0.83	0.83	0.83	0.83	0.83
	H_3_BO_3_	6.2	6.2	6.2	6.2	6.2
	ZnSO_4_	8.6	8.6	8.6	8.6	8.6
	Na_2_MoO_4_	0.25	0.25	0.25	0.25	0.25
	CuSO_4_	0.025	0.025	0.025	0.025	0.025
	CoCl_2_	0.025	0.025	0.025	0.025	0.025
	MnSO_4_	22.3	22.3	22.3	22.3	22.3
Iron salt (mM)	EDTA-Fe	0.05	0.05	0.05	0.05	0.05

### Sampling and Plant Growth Parameter Measurements

At the end of the experiment (after 5 weeks of treatment), plants of each treatment were harvested randomly, rinsed with deionized water, blotted carefully with tissue paper, and divided into leaf, stem, and root. Leaf area (cm^2^) was measured by a leaf area meter (Li-3100C; LI-COR Biosciences Inc., Lincoln, NB, United States). The fresh weight of shoots and roots were measured by an electronic analytical balance (FA, 2014), and the fresh shoot weight/fresh root weight (S/R) ratio was calculated. Seedling height (cm) and taproot length (cm) were measured using a scaled ruler. The representative roots and leaves from the different N treatments were imaged using a PowerShot Pro 1 camera (Canon, Tokyo, Japan).

Root activity was measured by the TTC method (Baozhang et al., 1994). Briefly, 0.5 g of the root tip samples, after being rinsed with deionized water were placed into a breaker containing 10 mL mixture solution with the same volumes of 0.4% TTC and phosphate buffer solutions. Roots were fully immersed in the solution and incubated at 37°C for 3 h under dark conditions. Then 2 mL of 1 mol/L sulfuric acid was added to stop the reaction. The roots were removed and blotted carefully with tissue paper. The formazan was extracted in 10 mL of ethyl acetate solution by grinding for 5 min with a mortar and pestle. Next, the homogenate was transferred into a 10 mL falcon tube and spun at 3,000 ×*g* for 10 min. The supernatant was transferred into a new 10 mL falcon tube. The optical density (absorbance) of the extract was measured at 485 nm with a UV-1800 spectrophotometer (AuCy, China). The root activity was calculated by the following formula: Reduction strength of tetrazolium [mg/g (root fresh weight)/h] = reduction amount of tetrazolium (mg)/[root weight (g) × time (h)].

### Analysis of Root System Architecture (RSA)

Seedlings of pear were randomly sampled in each treatment group and rinsed with deionized water before analysis. For the RSA analysis, root samples were scanned using an Epson digital scanner (Expression 10000XL 1.0, Epson Inc., Japan), and the root images were analyzed with WinRhizo software (Regent Instruments Canada Inc., 2013). Traits of total root length, total root surface area, total root volume, total root number per plant and average root diameter were calculated for each nitrate treatment.

### Chlorophyll Measurement

Chlorophyll was extracted from samples of 100 mg fresh leaves which were cut into small pieces with scissors and ground for 5 min in 10 mL of 85% acetone with a mortar and pestle. The homogenate was sieved through filter paper, transferred into a 15 mL Falcon tube, and adjusted to a set volume with 85% acetone. The absorbance of the extract was measured at both 663 and 644 nm with a UV-1800 spectrophotometer (AuCy, China). The concentrations of chlorophyll a and b, in mg per gram of FW sample, were calculated using the following formulae:

$$\begin{array}{l} \text{Milligrams chlorophyll a/g FW }=\;1.07\;({\text{OD}}_{663})-0.094({\text{OD}}_{644})\\ \text{Milligrams chlorophyll b/g FW }=\;1.77\;({\text{OD}}_{644})-0.280({\text{OD}}_{663})\\ \text{Total chlorophyll }=\text{ chlorophyll a }+\text{ chlorophyll b} \end{array}$$

### Nitrate Reductase Activity Measurement

For determination of nitrate reductase activity, leaves or roots harvested after the different N treatments were rinsed with distilled water, air-dried, and immersed in 10 mL of extraction buffer containing 1 mL 30% trichloroacetic acid, 4 mL 0.1 mol⋅L^-1^ phosphate buffer (pH 7.5) and 5 mL 0.2 mol⋅L^-1^ KNO_3_ for 30 min at 30°C in the dark. After 30 min, 1 mL 30% trichloroacetic acid was the added and the solution was agitated. The reaction was stopped by centrifugation at 6,000 ×*g* for 8 min. 2 mL of the supernatant was treated with 4 mL 1% sulfonamide and 0.2% α-naphthylamine before incubating for 20 min at 30°C in a water bath with agitation. Nitrate reductase activity was then measured at 540 nm using a UV-1800 spectrophotometer (AuCy, China). The level of nitrate reductase activity, the amount of nitrite produced in nmol per FW (g) root or foliage tissue per hour, was calculated by the following formula: NR activity (μg/g FW⋅h) = Nitrite content (μg) × sample dilution multiple/sample FW (g) × time (h).

### Elemental Analysis

The dried roots, shoots, and leaves (100–900 mg) were weighed into PTFE digestion tubes and digested by nitric acid with a microwave digester (Ultraclave 4; MLS). Elemental content analysis was undertaken by ICP optical emission spectroscopy (ICP 6500 dual OES spectrometer; Thermo Fischer Scientific, Waltham, MA, United States). For N analysis, the dried plants were frozen and ground in a ball mill. One mg of the ground material was taken for analysis using a Kjeldahl apparatus (JK9870).

### Experimental Design and Statistical Analyses

The experiment was established in a completely randomized 1 × 5 factorial design with one rootstock source subject to five N treatments ( 0, 4, 16, 32, and 64 mM N). Three replicates (three plants per replicate) were used for each treatment. Statistical analyses of the data were performed using the SPSS statistical package and the differences were statistically compared by employing the Duncan test with a significance level of *p* < 0.05.

## Results

### Visible Symptoms and Plant Growth

**FIGURE 1:**
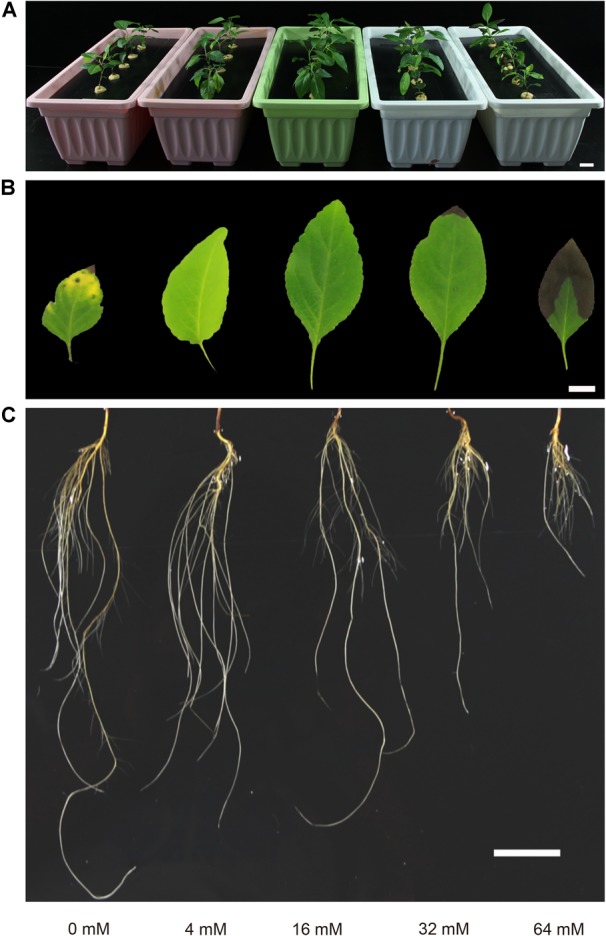
The effect of N supply on pear seedling growth and the production of visible symptoms. Planted seedlings are shown in panel **(A)**. Representative leaves and roots are depicted in panel **(B,C)**, respectively. The bars in each panel = 2 cm.

**FIGURE 2:**
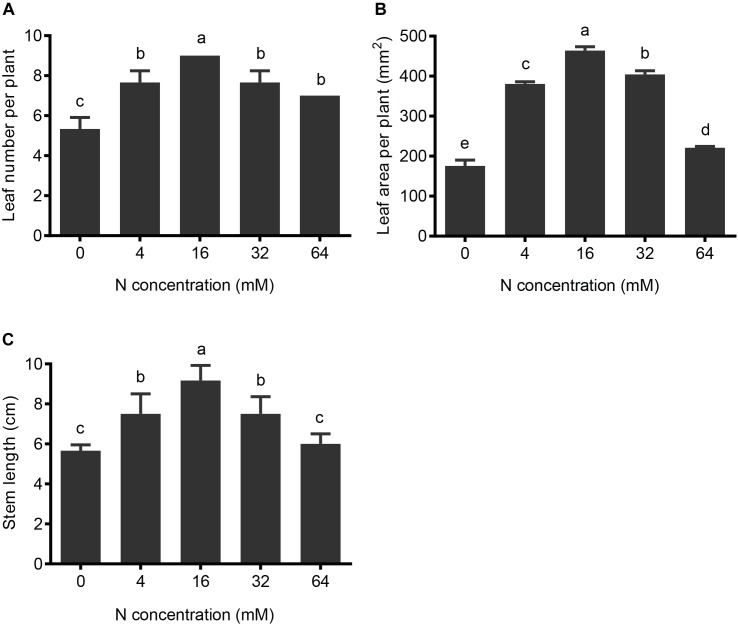
The effects of N treatments on pear shoot growth. **(A)** The leaf number per plant, **(B)** the leaf area per plant, and **(C)** the stem lengths were analyzed after different N treatments for 5 weeks. The data are presented as the means ± SDs of three replicates, and each replicate is based on measurements made from three plants. Letters in superscript indicate whether significant differences (*p* < 0.05) exist between different N treatments (differing letter) or not (same letter).

Significant variations were observed with different N treatments in our experiment. As shown in Figure 1, N deficiency and excess N treatments significantly influenced the growth and development of the pear seedlings. Seedlings displayed the best growth and development in the presence of 16 mM N, whereas extension of the stem and leaf expansion were significantly inhibited under N deficiency conditions (*p* < 0.05) (Figures 1A, 2B,C). Conversely, N deficiency stimulated the elongation of the root (Figure 1C). On the other hand, the growth of both the shoot and root were inhibited with excess N, especially in the root (Figure 1C). Since N can be transferred from old leaves to young leaves, N deficiency symptoms first appear in the older leaves, most of which discolor to light green-yellow and undergo early defoliation under severe N-deficiency conditions (Figure 1B), which are the typical symptoms of N deficiency in plants. Furthermore, the symptoms of the N deficiency in the leaf were reduced under conditions of moderate N deficiency (4 mM), where lighter yellow and larger leaves were exhibited relative to the severe N-deficient supply (Figure 1B). On the other hand, an excess supply of N induced symptoms different from those of N deficiency, i.e., a dark green leaf color accompanied by a scorch phenomenon, and these symptoms became more pronounced when the supply of N was increased (Figure 1B). In summary, N deficiency and excess N supply leads to the inhibition of pear seedling growth and development.

### Root Architectural Plasticity in Responses to N Treatments

In order to evaluate the effect of different levels N supply on the growth and development of the pear, the agronomic traits such as RSA, plant height, leaf number, leaf area, and physiological and biochemical traits such as mineral nutrient content, chlorophyll content, root activity, and nitrate reductase activity were further analyzed.

Consistent with the above studies, N-deficiency stimulated the growth of a more exploratory root system, which was longer and more highly branched (Forde and Lorenzo, 2001; Little et al., 2005; Luo et al., 2013). Detailed RSA analyses revealed that a low N supply not only stimulated LR length, but also increased the number of LR per plant relative to the control N treatment (16 mM), thereby significantly increasing the total plant root length, root surface area, and root volume (Table 2). Moreover, this stimulatory effect was markedly increased when further reducing the N supply from a moderate N deficiency (4 mM) to a severe N deficiency (0 mM; Table 2). However, the average root diameter showed no significant changes among these N treatments. On the other hand, the maximum root length, total root length, total root number, and total root surface area per plant showed a significant reduction in response to an excess supply of N. However, no significant difference was observed in the total root volume per plant between conditions of moderate and severe excesses of N treatments (Table 2).

**Table 2:** Effects of different N levels on root morphologic parameters of pear seedlings.

Treatments per plant (mM)	Root length (cm)	The max length of root (cm)	Root surface area (cm^2^)	Root volume (cm^3^)	Root number	Diameter (mm)
0	188.54^a^	25^a^	22.39^a^	0.17^a^	294^a^	0.3538^a^
4	166.42^b^	20.5^b^	17.14^b^	0.14^b^	158^c^	0.3501^a^
16	144.15^c^	16.67^c^	16.79^b^	0.1^c^	202^b^	0.3504^a^
32	80.62^d^	12.67^d^	13.31^c^	0.07^d^	144^c^	0.3665^a^
64	43.5^e^	7^e^	7.77^d^	0.06^d^	94^d^	0.4016^a^

*All pear seedlings were grown under hydroponic conditions with the nitrogen supplies indicated for 5 weeks. Data are presented as the mean ± SD of three replicates, and each replicate datum was derived from measurements from three plants.*

*Letters in superscript indicate whether mean values are significantly different between treatments (*p* < 0.05; different letters) or not (same letter).*

### The Effect of N Treatments on the Aerial Growth of Pear Seedlings

The shoots of pear seedlings are also highly sensitive to different N treatments. Both 4-mM N and 0-mM N-treated plants had significantly shorter heights and reduced leaf numbers and leaf areas, relative to control conditions (16 mM N). For the 4-mM N-treated plants, the leaf number, leaf area, and plant height decreased by 8% (Figure 2A), 18% (Figure 2B), and 18% (Figure 2C), respectively, while in the 0-mM N-treated plants, they decreased by 41% (Figure 2A), 62% (Figure 2B), and 38% (Figure 2C), respectively.

**FIGURE 3:**
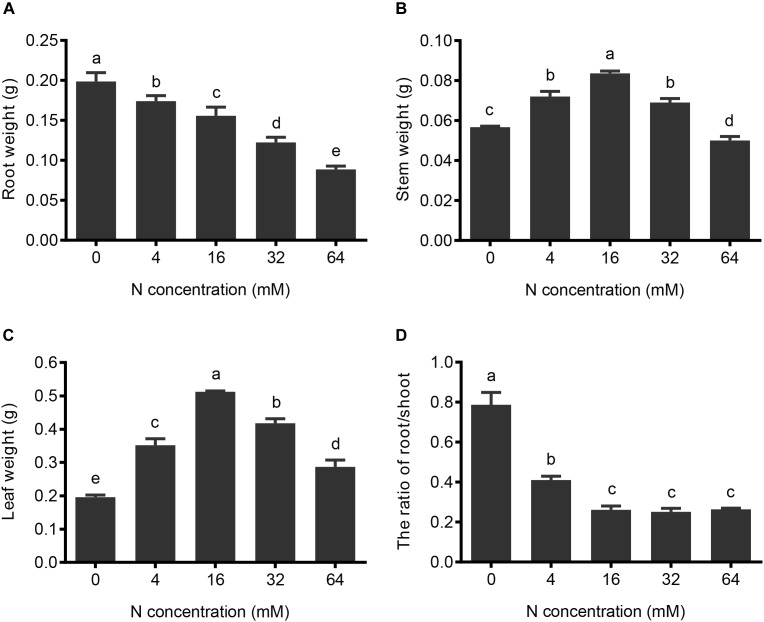
The effects of N treatments on the fresh weight of the different parts of the pear. **(A)** Root fresh weight, **(B)** stem fresh weight, and **(C)** leaf fresh weight under different N concentrations were determined. **(D)** Root fresh weight/shoot fresh weight ratio was calculated according to the results shown in **(A–C)**. The data are presented as described in Figure 2.

**FIGURE 4:**
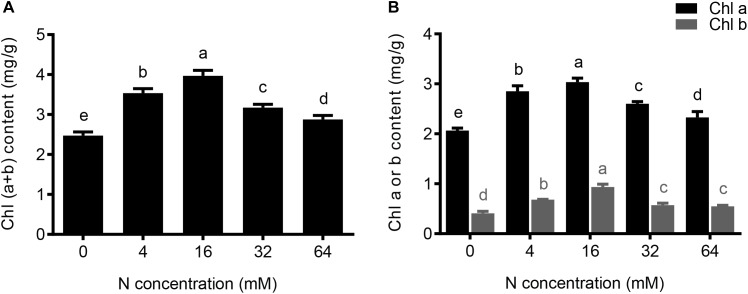
The effects of different N concentrations on the chlorophyll content of the pear seedlings. The data is presented as the means ± SDs of three replicates, and each replicate is based on measurements made from three plants. Different superscript letters indicate significant differences (*p* < 0.05) between different N treatments. **(A)** Total chlorophyll (a + b) content, **(B)** Chlorophyll a or chlorophyll b content under different N concentrations were determined.

### The Effect of N Treatments on the Ratio of Root/Shoot Fresh Weight

To evaluate the effects of the N deficiency and N excess on the growth and development of the pear seedlings, the fresh weights of roots, stems, and leaves grown under different N treatments were determined and the ratio of root/shoot weight was calculated. Since the length and number of roots increased from 64 to 0 mM N, the root fresh weight concomitantly increased. Compared to the 16 mM N control conditions, the 4 mM N and 0 mM N treatments resulted in a 16 and 43% root fresh weight increase, respectively (Figure 3A). On the other hand, because the growth of the stem and leaf were significantly suppressed under conditions of both N deficiency and N excess, the stem and leaf fresh weights initially increased with N supply from 0 to 16 mM, and then decreased at higher concentrations of supplied N (Figures 3B,C). Interestingly, leaf fresh weight appears to be more sensitive to changes in N conditions and demonstrates a significant difference between moderate N deficiency and a moderate excess of N supply, whereas no significant difference was found in stems under these conditions, suggesting leaves are more sensitive to changes in the N supply than stems. The ratio of root/shoot fresh weight mainly reflects the situation of the photosynthetic product distribution between the shoot and root (Figure 3D). The experimental results showed that roots displayed better growth than aerial portions of the plant under conditions of N deficiency, leading to a decrease in the root to shoot ratio when increasing the N supply from severe N deficiency to control (16 mM) conditions. However, no significant changes in the ratio could be detected between the control and higher (excess) levels of control supply, and this was mainly due to the fact that the growth of the shoot and root were both inhibited when further increasing the N supply.

### The Effect of N Treatments on Chlorophyll Concentrations

As shown in Figure1B, yellowing and dark green of the leaves were observed in pear seedlings under N deficiency and N excess conditions, respectively. In order to investigate the impact of N treatments on leaf chlorophyll, the leaf chlorophyll contents among five N treatment plants were examined. Comparisons of the data in Figure 4A showed that the total leaf chlorophyll content significantly decreased by 11 and 38% in 4- and 0-mM N-treated plants, respectively, compared with control (16 mM) plants. In addition, the synthesis of the total chlorophyll was also significantly inhibited with a moderate and severe excess in N supply, decreasing by 20 and 27% in the presence of the 32 and 64 mM N, respectively, compared to control plants (Figure 4A). Furthermore, the trends of chlorophyll a and chlorophyll b levels in response to the increasing ${\text{NO}}_{3}^{-}$ concentrations, were similar to that of total chlorophyll (Figure 4B). Taken together these results indicated that the inhibition effect induced by an excessive supply of N is between those observed under conditions of severe N deficiency and moderate N deficiency.

### The Effect of N Treatments on Root Activity

**FIGURE 5:**
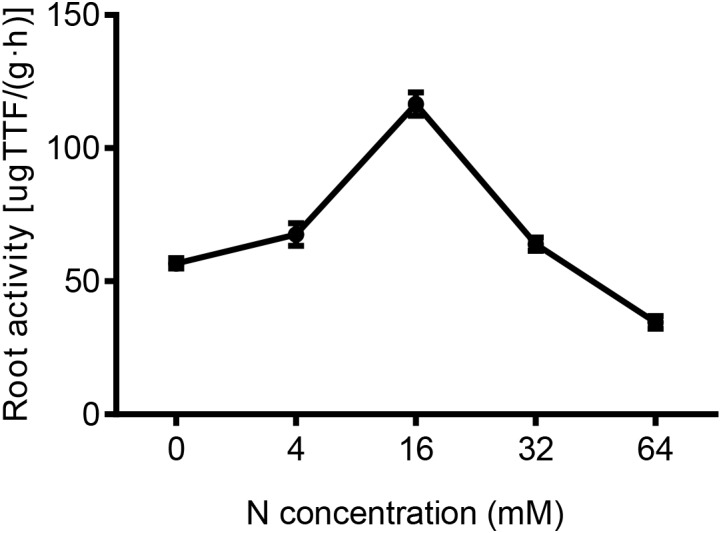
The effects of different N concentrations on the root activity of the pear seedlings treated for 5 weeks. The data is presented as the means ± SDs of three replicates (*n* = 3), and each replicate is based on measurements made from three plants.

To some extent, a strong root system activity is indicative of a vigorous root metabolism and absorption capacity. As shown in Figure 5, the root activity was markedly decreased in response to both N-deficient and N-excess treatments, whereas, relative to the control (16 mM N), it was decreased by 48 and 51% at 4 and 0 mM N, and by 45 and 70%, in 32 mM and 64 N-treated plants, respectively. Taken together these results suggested that the root activity was severely inhibited by N deficiency and an excess supply of N.

### Effects of Different Nitrate Levels on Nitrate Reductase Activity in Pear Seedlings

Nitrate reductase is one of the key enzymes in plant N metabolism and assimilation process. Its activity determines the rate of assimilating nitrate into organic N. Nitrate reductase is an inducible enzyme whose abundance is affected by the exterior concentration of nitrate. Therefore, we investigated the effects of different concentrations of exogenous nitrate ions on the nitrate reductase activity in leaves and roots of pear seedlings.

**FIGURE 6:**
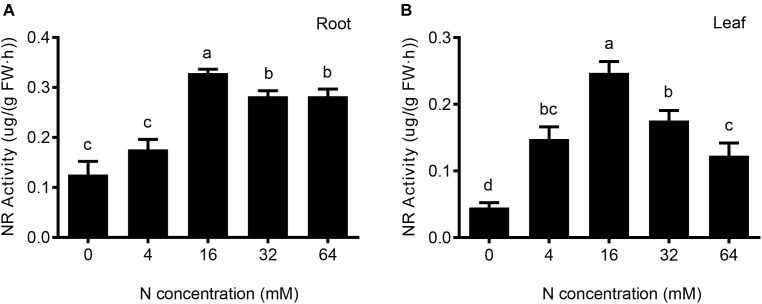
The effects of different N levels on the nitrate reductase activity of the root **(A)** and leaf **(B)** were analyzed. The level of nitrate reductase activity was represented by the amount of nitrite produced in nmol per FW (g) of root or foliage tissue per h. The data are presented as the means ± SDs of three replicates, and each replicate is based on measurements made from three plants. Different superscript letters indicate significant differences (*p* < 0.05) exist between different N treatments.

As shown in Figure 6A, N deficiency and N excess treatments significantly reduced the nitrate reductase activity in pear root; moreover, the inhibitory effect under conditions of N deficiency is greater than that under conditions of excess N. However, no significant difference was detected between conditions of severe and moderate N deficiency or between severe and moderate excess of N. The nitrate reductase activity in leaves is also clearly influenced by the different N treatments (Figure 6B). The results show that significant differences were observed among these five N treatments, the nitrate reductase activity in leaf decreased compared with root under both of deficient and excessive N conditions.

### Mineral Element Contents in Different Seedling Organs

**FIGURE 7:**
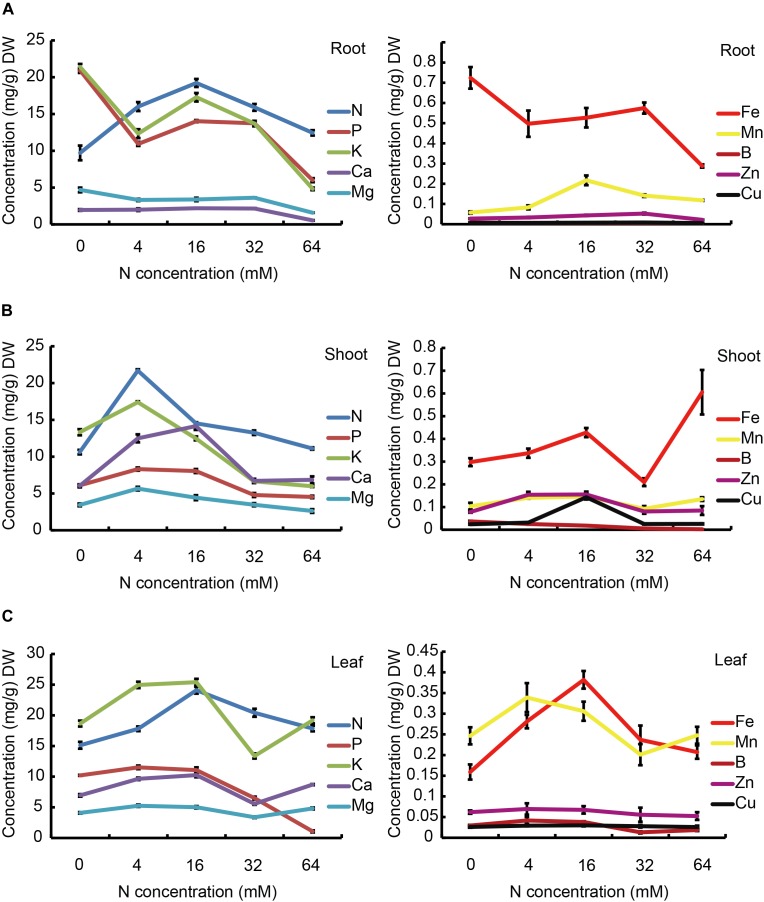
The effects of N treatments on the macroelement and microelement contents of the root **(A)**, stem **(B)**, and leaf **(C)**. The data are presented as the means ± SDs of three replicates (*n* = 3), and each replicate is based on measurements made from three plants.

The concentrations of mineral macronutrients (N, P, K, Ca, and Mg) and micronutrients (Fe, Mn, B, Zn, and Cu) in roots, stems, and leaves of pear seedlings treated with five levels of N are presented in Figure 7. The endogenous N concentration in the roots gradually increased with increasing N supply when the level of N supply was less than 16 mM, but significantly decreased when the N supply was over 16 mM (Figure 7A, left panel). It is worth noting that in pear root, compared with the N content, the P and K contents showed different trends when the plants were grown in an N-deficient (0 or 4 mM) environment. They showed a significant decrease when the N supply increased from 0 to 4 mM. However, the P and K contents showed similar trends to that of the N when the N supply increased from 4 to 64 mM. Ca^2+^ and Mg^2+^ levels showed no significant differences under the N concentration increased from 4 to 32 mM, while a severe excess N supply markedly reduced the concentrations of Ca^2+^ and Mg^2+^. It is interesting that the Mg concentration was markedly decreased when increasing the N supply from 0 to 4 mM. As shown in Figure 7A (right panel) not only the macronutrient concentrations, but also the micronutrients concentrations were influenced by different N treatments. The concentration of Fe displayed a similar trend to P and K, which is markedly increased in the presence of 0 mM N, but showed a significant decrease under severe N-excess conditions. In addition, the concentration of Mn displayed a different trend to Fe, showing a significant decrease under both N deficiency and excess treatments. Interestingly, B and Cu were hardly detected in the root.

Different trends were observed for both the macronutrients and micronutrients in the stem with different N treatments when compared to those displayed in the root (Figure 7B). It is worth noting that the maximal concentrations of all macronutrients, except for Ca, were obtained at 4 mM N, but not at 16 mM N. As for the micronutrients, the contents of Fe, Mn, and Zn showed a similar trend in response to different N treatments, which was of a significant increase when increasing the N supply from 0 to 4 mM, but B and Cu showed no significant changes. Moreover, the concentrations of Fe and B displayed a significant increase when further increasing the N supply from 4 to 16 mM, but no significant changes were observed in Mn and Zn. In addition, the concentrations of most micronutrients displayed an obvious decrease when further increasing the N supply to 32 mM, whereas no effects was observed on the concentrations of Mn and Zn. Importantly, all micronutrients were significantly reduced in the presence of 32 mM N and most displayed no significant changes at the higher concentration of 64 mM N. Conversely, Fe and Mn showed marked increases in response to this increase in N.

The concentrations of the macronutrients and micronutrients in the leaf were also sensitive to different N treatments, as shown in Figure 7C, the concentrations of N were largely increased with increasing the N supply from 0 to 16 mM, but P, K, Ca and Mg were significantly increased when increasing the N supply from 0 to 4 mM, but not influenced by further increasing the N supply to 16 mM. In the case of excess N, the concentrations of P and N were significantly decreased, but the concentrations of K, Ca, and Mg displayed first a reduction and then an increase. Fe showed a similar trend to N, while Mn exhibited a similar trend to K. However, the concentrations of leaf Zn and Cu were not influenced by the N treatments. Furthermore, the concentration of B was markedly decreased with an excess supply of N, but not affected by N-deficiency.

## Discussion

### Symptoms and Plant Growth

Several studies have shown that changes in N supply can induce physiological phenotypic changes in the plants (Gruber et al., 2013; Canales et al., 2014). In poplars, N deficiency induced root biomass production and inhibited the growth of shoots and leaves, whereas a high N supply repressed root growth and reduced shoot biomass (Luo et al., 2013; Euring et al., 2014). Studies in wheat also demonstrated that the root length was increased while the shoot length was reduced under N-starvation conditions (Sinha et al., 2015). Consistent with previous results, the N deficiency caused the yellowing of mature leaves, and total root number and the fresh root weight were significantly increased under N-deficiency conditions, but stem biomass, leaf number, leaf area and leaf biomass were markedly decreased. However, the excessive N treatment caused dark green leaves accompanied by coking, and root elongation, shoot extension and leaf expansion were all suppressed by the excessive N application. Thus, these results indicated that a moderate N fertilizer supply is crucial to ensure normal growth and development in pear trees.

### Relationship Between Physiological Performance and Mineral Nutrient Contents

Previous research showed that mineral content can provide information regarding the functional state of organisms under different growth conditions (Thornton, 2002). Ionomics has been widely used in many plant mineral nutrient studies, such as in tomato (Sanchez-Rodriguez et al., 2010), rice (Norton et al., 2010), soybean (Wang et al., 2012), and grapevine (Sofo et al., 2013). Here, the mineral nutrient concentrations in the leaf, stem and root of the pear rootstocks exposed to different concentrations of N were analyzed to explain the symptoms of the pear under N-stress conditions. Our results showed that not only endogenous N concentrations but also other mineral nutrient concentrations in the roots, stems and leaves of pear seedlings were influenced by N-deficiency and N-excess treatments. It may be that N has not only synergistic effects on other mineral elements, but also forms antagonistic effects, affecting other mineral elements’ absorption, distribution or utilization in plants, and finally determining other mineral elements’ contents in different plant organs (Feil and Banziger, 1993). It may also be that root activity is markedly decreased by N-deficiency and N-excess treatments, which then affects other mineral elements’ absorption. For example, as shown in Figure 7C, the N content in the leaves significantly decreased under N-deficiency conditions, whereas Mg levels appeared insignificantly changed when reducing the N supply from normal to moderate N-deficiency. However, leaf Mg content was significantly decreased under severe N-deficiency conditions. Because N is an important component of chlorophyll, and Mg occupies a central position in the chlorophyll molecule and enzymatic cofactor (Maathuis, 2009). We concluded that leaf yellowing under moderate N-deficiency conditions could be attributed to the decrease in N concentration, while severe leaf yellowing in the presence of 0 mM N might be attributed to a decrease in the concentrations of N and Mg. Additionally, Fe and Mn are essential elements for leaf chlorophyll metabolism and photosynthesis (Hansch and Mendel, 2009). Here, the leaf Fe and Mn contents were significant decreased under N-deficiency conditions (Figure 7C). Thus, leaf yellowing in pear under N-deficiency conditions may be caused by decreased concentrations of N, Mg, Fe, and Mn, which might affect the photosynthetic rate of pear.

In addition, the ionomic analysis indicated that a high N supply significantly reduced the contents of P, K, Mg, Fe and Mn ions in the leaf, but the contents of K, Mg, and Mn under 64-mM N conditions were greater than those at 32 mM N. It may be that the growth of the leaves was severely suppressed, leading to a concentration-dependent N effect. However, the P and Mn concentrations in the leaf significantly decreased as the N supply increased from 16 to 64 mM. The decrease in P concentration in particular leads to the typical symptoms of P deficiency, as indicated by the dark green leaves. Moreover, a shortage of K results in the leaf edges coking. Thus, an excessive N supply appears to induces a shortage of P and K in pear leaves, contributing to the dark green color and coking.

### Possible Mechanisms That Underlie the Morphological and Physiological Phenotypes

Higher root activity values indicate stronger root metabolic activities and absorption capacities (Haluskova et al., 2009). Our results showed that root activity was both inhibited under low and excessive N supply, but the inhibitory effect was more obvious under excess N than under N-deficiency conditions. Based on the results mentioned above, we may conclude that N deficiency and N excess not only influenced the elongation of the pear root, but also suppressed root activity in pear. Additionally, nitrate reductase, a key enzyme in plant N assimilation and metabolism (Hirel et al., 2001), is also significantly reduced under N-deficiency and N-excess conditions. Thus, physiological phenotype correlated with changes in the biochemical indexes.

The molecular mechanism that underlies morphological and physiological acclimation under N starvation and excess has also been widely studied. It was reported that nitric oxide (NO) was considered a key regulator for root growth under conditions of N-deficiency, where NO, together with strigolactones, may be involved in regulating IAA transport from the shoot to the root, leading to increases in the root number and seminal root-tip elongation through promotion of increased root meristem activity (Sun et al., 2014; Manoli et al., 2016). However, in the presence of high concentrations of ${\text{NO}}_{3}^{-}$-N nutrient solution will continue to stimulate the increase of cytokinin content, antagonize IAA, significantly reduce the IAA content (Caba et al., 2000; Walch-Liu et al., 2006) and promote the production of ethylene and ABA (Signora et al., 2001) in roots, thereby inhibiting the growth of roots (Tian et al., 2008, 2009). This may be the main reason for the inhibited root growth of pear seedlings under high N-treatment conditions in this experiment. Furthermore, other studies showed highly significant negative correlations between the leaf nitrate content and total root growth (Marriott and Dale, 1977), suggesting that nitrate in the leaf may act as a signal, reflecting internal N status and mediating root growth. Whether these mechanisms mentioned above are applicable to the pear seedlings under conditions of N deficiency and N excess needs to be further investigated.

## Author Contributions

GC designed the research, performed the experiments, and analyzed the results. LW drafted the manuscript. YT, PC, and QC participated in carrying out the experiments. KQ, SZ, and ST participated in revising the final manuscript. JW managed the experiments. All authors have read and approved the final manuscript.

## Conflict of Interest Statement

The authors declare that the research was conducted in the absence of any commercial or financial relationships that could be construed as a potential conflict of interest.

## Abbreviations

ABA: abscisic acid
IAA: auxin
ICP: inductively coupled plasma
LR: lateral root
N: Nitrogen
RSA: root system architecture
TTC: triphenyl tetrazolium chloride
